# Observations on the Management of Madhouses. Part II. Containing an Account of Susannah Roginson, &c.

**Published:** 1841-04

**Authors:** 


Art. XII.—*Observations on the Management of Madhouses.* Part II. *Containing an Account of Susannah Roginson, &c.* By Caleb Crowther, m.d., formerly Senior Physician to the West Riding Pauper Lunatic Asylum.—*London*, 1841. 8vo, pp. 104.

The venerable author of this little work has laid open, with unsparing hand, the alleged negligences of some of the institutions in his neighbourhood; and has forcibly described the qualities desirable in the superintendent of a lunatic asylum. He alludes, in terms of high commendation, to the general management of the large asylum at Hanwell; and his testimony to the zeal, industry, and benevolence of its physician, Dr. Conolly, may be taken as a well-timed set-off to the part taken by a small minority of the magistrates of Middlesex at the late meetings at Clerkenwell. Many of Dr. Crowther's remarks deserve attentive consideration, both from magistrates and physicians. He is very severe upon those whose proceedings he views with disapprobation; but some of the facts stated by him are of a very startling kind, and remind us of similar enormities in another asylum in Yorkshire, which were long denied, long concealed, long protected by great authorities, and proved at last. It is remarkable that Dr. Crowther's charges apply chiefly to an asylum of which the directors seem to pride themselves on keeping the beaten track, and resisting all improvement. Any further notice of this work would engage us in angry discussions in which we have no desire to take a part: but if the facts related by Dr. Crowther are correct, they ought surely to be looked into.

